# Leader Forgiveness and Employee’s Unethical Pro-organizational Behavior: The Roles of Gratitude and Moral Identity

**DOI:** 10.3389/fpsyg.2021.698802

**Published:** 2021-08-26

**Authors:** Lu Lu, Yuchu Huang, Jia Luo

**Affiliations:** School of Business Administration, Guizhou University of Finance and Economics, Guiyang, China

**Keywords:** leader forgiveness, gratitude, unethical pro-organizational behavior, moral identity, moderated mediation model

## Abstract

Leader forgiveness refers to the abandonment of anger, resentment, and the desire to revenge against the offender, and it not only means forgiving errors or mistakes made by employees, but also means empathizing and understanding employees, and to see things from another point of view. This research examines the possible “dark side” of leader forgiveness by examining its influence on employee’s unethical pro-organizational behavior, as well as the mediating effect of gratitude and the moderating effect of moral identity. We used questionnaire survey methodology to collect data from 263 Chinese employees to test our hypotheses. Results show that leader forgiveness had a positive influence on employee’s unethical pro-organizational behavior, and gratitude mediated the influence of leader forgiveness on unethical pro-organizational behavior. The relationship between gratitude and unethical pro-organizational behavior, and the indirect influence of leader forgiveness on unethical pro-organizational behavior through gratitude, were moderated by moral identity. Theoretical and practical implications are discussed.

## Introduction

As workplace environment becoming “toxic” in nowadays (Rasool et al., 2019a), conflict has become a pervasive phenomenon in organizations. Thus, when employees conflict with their leaders regarding opinions and decisions, or do something wrong, it is important that leaders forgive and try to repair their relationships. In academic circles, leader forgiveness, defined as the abandonment of anger, resentment, and the desire to revenge against the offender (Aquino et al., 2006), has also drawn a growing body of interest from researchers, and is suggested to be a critical value of the modern leader (Caldwell and Dixon, 2010). Leader forgiveness not only means forgiving errors or mistakes made by employees, but also means empathizing and understanding employees, and to see things from another point of view (Rodríguez-Carvajal et al., 2014).

Previous research regarding leader forgiveness has covered many positive influences of leader forgiveness on employees, such as increasing employees’ physical and mental health, job satisfaction, trust and sense of belonging to the leader, and cultivating high-quality relationship between leaders and employees (Fincham and Beach, 2007; Karremans and Van Lange, 2008; Bies et al., 2016). However, despite existing research have laid an important foundation for exploring the influence of leader forgiveness on employees’ positive behaviors, they have neglected that forgiveness may lead to undesired consequences and be detrimental (Fehr and Gelfand, 2012; Adams et al., 2015). Therefore, when and how does leader forgiveness lead to negative outcomes? These questions remain unanswered in the existing literature.

In the current research, we propose that when leaders forgive employees’ errors or mistakes, employees will consider it as an emotional support from leaders. Based on the principle of reciprocity of social exchange, in the process of exchange, one will feel responsible to return after receiving help or support from another (Cropanzano and Mitchell, 2005), and under such obligation of return, employees often participate in extra-role behaviors. In addition, by forgiving, employees may feel that leaders will tolerate and not punish their mistakes as long as they have beneficial intentions, thus they are likely to conduct pro-organizational behaviors that violate ethical norms. One of such behaviors could be unethical pro-organizational behavior, which refers to the unethical behavior of employees in order to promote the effective operation of the organization or its members, but violate core social values, morals, laws or standards (Umphress and Bingham, 2011).

Based on affective event theory (Weiss and Cropanzano, 1996), workplace events can trigger employees’ emotional responses, and then affect their subsequent attitudes and behaviors. Since leader attitude and behavior is often one of the most important events in the workplace, leader forgiveness could also cause employees’ emotional response. Research has found that employees with gratitude will embrace this feeling for their work and make efforts to return to the organization (Fehr et al., 2017). Since leader forgiveness reflects empathy and tolerance, which will make employees feel the concern and warmth of the leader or the organization, which may trigger employees’ feelings of gratitude. Therefore, we incorporate gratitude, a response to the benevolent behavior of others, to examine whether leader forgiveness leads to unethical pro-organizational behavior by triggering employees’ feelings of gratitude.

Moreover, it is possible that not all employees will be promoted by gratitude and go beyond the moral bottom line to conduct unethical behaviors for the benefit of organizations. In other words, individual differences could be a critical conditional factor that determines whether employees with gratitude will conduct unethical pro-organizational behavior. Moral identity reflects how important and significant that moral values in one’s self-concept (Aquino and Reed, 2002), such that high moral identity individuals tend to behave in ethical ways to be consistent with their moral selves (O’Reilly and Aquino, 2011). Therefore, this article speculates that moral identity may be an important moderating role in the relationship between gratitude and unethical pro-organizational behavior.

Based on the above analysis, this study proposes the following research questions:

RQ1: Does leader forgiveness lead to employees’ unethical pro-organizational behavior?

RQ2: Does gratitude mediate the relationship between leader forgiveness and unethical pro-organizational behavior?

RQ3: How does moral identity moderate the relationship between gratitude and unethical pro-organizational behavior?

The following part of this paper is organized as follows: Section 2 presents hypotheses development and research model. Section 3 presents research methods and results. Section 4 provides discussion, theoretical implications, practical implications, limitations and future research directions. Second 5 is the conclusion of the study.

## Theories and Hypotheses

### Leader Forgiveness and Unethical Pro-organizational Behavior

Umphress and Bingham (2011) have proposed a special unethical behavior, unethical pro-organizational behavior, and defined it as “actions that are intended to promote the effective functioning of the organization or its members and violate core societal values, mores, laws, or standards of proper conduct” (p. 622). Based on their definition, unethical pro-organizational behavior is a complex of both unethical and pro-organizational components. Previous research has found that when employees feel psychological entitlement (Lee et al., 2019), perceive interpersonal injustice (Bryant and Merritt, 2021), or identified with the organization (Chen et al., 2016), they are more likely to conduct unethical pro-organizational behavior. Leader behaviors, such as ethical leadership (Miao et al., 2013), responsible leadership (Cheng et al., 2019), self-sacrificial leadership (Yang et al., 2020), and abusive supervision (Guo et al., 2020) could also influence employee’s unethical pro-organizational behavior.

Forgiveness in organizations is to foster employees’ abandonment of the resentment and blame, and to adopt positive and forward-looking behaviors to deal with the harm and damage caused by the conflict (Cameron and Caza, 2002). Leader forgiveness refers to leader’s choice to accept and to look past employee’s errors or mistakes, and has been acknowledged as a valuable quality of an effective leader (Caldwell et al., 2009). Forgiveness is also critical to repairing leader-follower relationship and helps to create a culture of trust, cooperation and commitment (Ren and Gray, 2009).

We expect leader forgiveness to impact unethical pro-organizational behavior for the following two reasons. First, in forgiving, leaders show empathy and trust, free employees from the burden of mistakes, and can benefit the self-esteem of employees. Thus, in the eyes of the forgiven, forgiveness suggests a favor from leaders. Based on social exchange theory, leader forgiveness offers emotional support to employees, and to repair their relationships with leaders, employees will put effort to share worries of the leader, solve the problems that affect the development of the organization, and actively play a beneficial role in the organization. Under this strong motivation to maintain and promote the interests of the organization, employees may even cross the boundaries of ethics. In other words, employees may even get rid of the restraint of morality on self-behavior to implement unethical pro-organizational behavior in order to reciprocate leaders.

Second, leader forgiveness usually helps to cultivate a safe culture where taking risks could be tolerant or even encouraged. On the other side, however, in the eyes of the forgiven employees, forgiveness suggests tolerance and empathy from leaders. In other words, in environment where forgiveness takes place employees are more likely to proactively conduct rule-breaking and unethical behaviors, as long as these behaviors are beneficial to the organization, since they expect that leaders will forgive. As regarding to unethical pro-organizational behavior, though it is unethical in nature, employees conduct unethical pro-organizational behavior for the purpose to benefit the organization and its members. Thus, it is reasonable to speculate that leader forgiveness could encourage employees’ unethical pro-organizational behavior. Therefore, we propose the following hypothesis:

Hypothesis 1. Leader forgiveness will be positively related to employee’s unethical pro-organizational behavior.

### The Mediating Role of Gratitude

Gratitude is defined as a feeling of thankfulness in response to an experience that is beneficial, but not attributable to, the self (Emmons and McCullough, 2004; Fehr et al., 2017). Previous research has suggested that gratitude is positively associated with individual’s positive emotions, well-being, and health (Seligman et al., 2005).

Based on affective event theory (Weiss and Cropanzano, 1996), employees’ behavior and attitude in the workplace are mainly determined by their emotional changes, and these emotions are often caused by situation factors or workplace events. For example, leaders usually hold more power, evaluate employees’ performance and interact with employees more often, thus act as a key role in influencing employees’ emotions. When employees commit offensive behaviors toward their leaders, they usually receive negative results such as demotion, salary cuts, and even expulsion. However, if the leaders choose to forgive employees for their negative behaviors, this will bring great benefits to employees. And the basic condition for generating gratitude is the benefits and favors that others give to individuals. Thus, leader forgiveness may lead to employees’ feeling of gratitude.

In addition, gratitude is not only to accept the beneficiaries of others, but also an important motivating factor to implement positive behaviors to give back to the beneficiary (McCullough et al., 2001). When employees generate gratitude after receiving forgiveness from leaders, this emotion would lead them to perform behaviors that are beneficial to the organization. Therefore, with the feeling of gratitude, employees would have a positive working attitude and devote a lot of energy to the organization, and when they are eager to repay the leaders or the organization, they may even neglect the unethical components of their behaviors. In other words, employees who are gratitude for leader forgiveness may conduct unethical pro-organizational behavior. Thus, we propose the following hypothesis:

Hypothesis 2. Gratitude will mediate the relationship between leader forgiveness and unethical pro-organizational behavior.

### The Moderating Role of Moral Identity

Moral identity describes the significance of moral values to an individual’s self-concept (Aquino and Reed, 2002). In other words, moral identity reflects to what extent individuals think that becoming a moral person and conduct ethical behavior is important to themselves. Because of the desire to be a moral person and regard moral identity as an important component of self-concept, individuals with high moral identity can quickly and lastingly activate their awareness of morality (Aquino et al., 2009). Previous research has also found that high moral identity could lead to pro-social behaviors (Reed et al., 2007), and low moral identity could increase individual’s unethical behaviors (Detert et al., 2008; Gino et al., 2011).

In this study, we propose that moral identity, as an individual difference in adjusting one’s own ethical behavior, acts as a moderating role in the relationship between gratitude and unethical pro-organizational behavior. Specifically, high moral identity individuals usually have higher requirements on their own morality, and their behavior is largely judged and restricted by their own moral standards, while individuals with low moral identity have lower requirements on their own moral standards, and when they engage in unethical behavior, they often reasonably explain their behaviors to reduce the responsibility of unethical behavior that may bring bad results. Therefore, when moral-related events occur, self-interest is more likely to be stimulated for low moral identity employees, conversely, those high moral identity employees could have the ability to regulate their behaviors. Thus, when employees feel grateful for leader forgiveness, compared with low moral identity employees, those with high moral identity will realize that only by doing appropriate ethical behaviors can they bring real benefits to the organization, and fulfill the expectations and support from leaders. On the contrary, for employees with low moral identity, when they have gratitude for leader forgiveness, they pay more attention to the beneficiary purpose of unethical pro-organizational behavior while are not sensitive to its unethical component. Therefore, we propose the following hypothesis:

Hypothesis 3. Moral identity will moderate the relationship between gratitude and unethical pro-organizational behavior, thus the relationship between gratitude and unethical pro-organizational behavior will be weaker when moral identity is high.

Based on the above analysis, leader forgiveness will influence employee’s unethical pro-organizational behavior through the mediating role of gratitude (Hypothesis 2), while moral identity moderates the relationship between gratitude and unethical pro-organizational behavior (Hypothesis 3). The above hypothesis lays the theoretical foundation of the moderated mediation model, that is, moral identity moderates the indirect influence of leader forgiveness on unethical pro-organizational behavior through gratitude. Specifically, when moral identity is high, the indirect effect of leader forgiveness on unethical pro-organizational behavior through gratitude will be weakened. Accordingly, the following hypotheses are proposed:

Hypothesis 4. The indirect effect of leader forgiveness on employee’s unethical pro-organizational behavior *via* gratitude will be moderated by moral identity, thus the indirect effect will be weaker when moral identity is high.

## Materials and Methods

### Research Approach

In this research, we followed the research approach adopted by other researchers (Rasool et al., 2019b; Zhou et al., 2021) and used a questionnaire survey to collect data. We adopted this approach for the following reasons: First, questionnaire survey method is a commonly used methodology in management research and enable us to collect a large number of reliable samples to test hypotheses. Second, the aim of our research is to examine how employees react to their perception of leader forgiveness, which is subtle, and unethical pro-organizational behavior is a type of hidden behavior, which is usually hard to be observed by others, thus it is reasonable to adopt self-reported questionnaires to measure these variables.

### Questionnaire Design

Three control variables (gender, age, education) and nineteen items were included in the questionnaire. Specifically, leader forgiveness was measured with five items (McCullough et al., 1997), gratitude was measured with three items (McCullough et al., 2002), unethical pro-organizational behavior was measured with six items (Umphress et al., 2010), and moral identity was measured with five items (Aquino and Reed, 2002). As regarding the control variables, in “Gender,” 1 = male; 2 = female; in “Age,” 1 = 25 years old and below, 2 = 26 to 35 years old, 3 = 36 to 45 years old, 4 = 46 years old and above; in “Education,” 1 = College degree and below, 2 = Bachelor degree, 3 = Master degree, 4 = Doctoral degree. All the measures were established scales adopted from related literature and were anchored on a five-point Likert scale ranging from 1 (strongly disagree) to 5 (strongly agree). As our research was conducted in China, we followed the translation and back-translation procedure (Brislin, 1986) to translate all English measures into Chinese.

### Samples and Procedures

We collected data from eight Chinese companies located in Beijing, Shanghai, Chongqing, Guizhou and other places. Employees reported leader forgiveness, gratitude, moral identity, and unethical pro-organizational behavior. 360 questionnaires were distributed in this survey, and 263 valid questionnaires were recovered. The effective response rate of questionnaires was 73%. Among employees participating in this survey, 53.2% are male and 46.8% are female; in terms of age, 22.8% are aged 25 and below, 41.8% are aged between 26 and 35, 19.0% are aged between 36 and 45, 16.4% are aged 46 and above, in terms of education, 13.3% with a high school degree and below, 22.4% with a college degree, 51.7% with a bachelor degree, 12.6% with a postgraduate degree. Table 1 shows the demographic characteristics of the samples in this study.

**TABLE 1:** Demographics of the respondents.

**Measure**	**Items**	**Frequency**	**Percentage (%)**
Gender	Male	140	53.2
	Female	123	46.8
Age	25 or below	60	22.8
	26–35	110	41.8
	36–45	50	19.0
	46 or above	43	16.3
Education	College degree and below	35	13.3
	Bachelor degree	59	22.4
	Master degree	136	51.7
	Doctoral degree	33	12.5

### Measures

#### Leader Forgiveness

A five-item scale adapted from McCullough et al. (1997) was used to measure leader forgiveness. A sample item was “My leader has forgiven my mistakes.” Cronbach’s alpha was 0.85.

#### Gratitude

A three-item scale adapted from McCullough et al. (2002) was used to measure employee’s feeling of gratitude. A sample item was “I feel grateful to my leader.” Cronbach’s alpha was 0.87.

#### Unethical Pro-Organizational Behavior

A six-item scale developed by Umphress et al. (2010) was used to measure employee’s unethical pro-organizational behavior. A sample item was “If needed, I would conceal information from the public that could be damaging to my organization.” Cronbach’s alpha was 0.95.

#### Moral Identity

A five-item scale developed by Aquino and Reed (2002) was used to measure employee’s moral identity. Participants were instructed to identify how important those characteristics to them, a sample item was “I strongly desire to have these characteristics.” Cronbach’s alpha was 0.93.

#### Control Variables

We controlled employee’s gender, age and education in this study.

## Results

### Confirmatory Factor Analysis

We used AMOS 22.0 to conduct confirmatory factor analysis to examine the discriminate validity of all variables in this study. As shown in Table 2, the four-factor model (χ2 = 288.649, df = 146, χ2/df = 1.977, CFI = 0.962, TLI = 0.955, RMSEA = 0.061) fits the data better as comparing with other models, suggesting that all variables have a good discriminate validity.

**TABLE 2:** Results of confirmatory factor analysis.

**Models**	**χ*^2^***	***df***	**χ*^2^*/*df***	***Δ*χ^2^ (*Δdf*)**	**CFI**	**TLI**	**RMSEA**
Four-factor model	288.65	146	1.98	—	0.96	0.96	0.06
Three-factor model: LF + GR	642.22	149	4.31	353.57***(3)	0.87	0.85	0.11
Two-factor model: LF + GR, MI + UPB	1781.45	151	11.80	1492.80***(5)	0.56	0.51	0.20
Single-factor model: LF + GR + MI + UPB	2299.97	152	15.13	2011.32***(6)	0.43	0.35	0.23

***p < 0.001. UPB, unethical pro-organizational behavior; LF, leader forgiveness; GR, gratitude; MI, moral identity; CFI, comparative fit index; TLI, Tucker–Lewis index; RMSEA, root mean square error of approximation.

### Descriptive Statistics and Correlation Analysis

The means, standard deviations and correlates were shown in Table 3.

**TABLE 3:** Means, standard deviations, and intercorrelations for study variables.

**Variable**	**M**	**SD**	**1**	**2**	**3**	**4**	**5**	**6**
1. Gender	1.468	0.500						
2. Age	2.289	0.996	–0.065					
3. Education	2.635	0.867	0.131*	–0.081				
4. Leader forgiveness	4.223	0.604	0.015	−0.156*	0.127*			
5. Gratitude	3.781	0.626	–0.061	0.073	0.110	0.410**		
6. Moral identity	3.129	1.133	0.019	–0.025	–0.084	–0.056	–0.065	
7. UPB	3.881	0.815	–0.094	0.056	0.069	0.222**	0.425**	0.111

N = 263. *p < 0.05 and **p < 0.01. In “Gender,” 1 = male; 2 = female; in “Age,” 1 = 25 years old and below, 2 = 26–35 years old, 3 = 36–45 years old, 4 = 46 years old and above; in “Education,” 1 = College degree and below, 2 = Bachelor degree, 3 = Master degree, 4 = Doctoral degree.

### Hypothesis Testing

We used hierarchical regression analysis and PROCESS Macro to test the hypotheses. As shown in Table 4, leader forgiveness was positively related to employees’ unethical pro-organizational behavior (model 4, β = 0.23, *p* < 0.001). Thus, hypothesis 1 was supported.

**TABLE 4:** Results of regression analysis.

**Variables**	**Gratitude**		**Unethical pro-organizational behavior**				
	**Model**	**Model**	**Model**	**Model**	**Model**	**Model**	**Model**
	**1**	**2**	**3**	**4**	**5**	**6**	**7**
Gender	–0.07	–0.07	–0.10	–0.10	–0.07	–0.07	–0.07
Age	0.08	0.14*	0.06	0.09	0.02	0.04	0.03
Education	0.13*	0.08	0.09	0.06	0.04	0.03	0.06
Leader forgiveness		0.42***		0.23***		0.07	
Gratitude					0.42***	0.39***	0.39***
Moral identity							0.19**
Gratitude × Moral identity							−0.16**
F	2.13	15.76	1.64	4.81	14.80	12.06	12.79
R^2^	0.02	0.20***	0.02	0.07**	0.19***	0.19***	0.23***
Δ R^2^	—	0.17***	—	0.05***	0.17***	0.12***	0.04**

N = 263. *p < 0.05, **p < 0.01, and ***p < 0.001.

In addition, leader forgiveness was positively related to gratitude (model 2, β = 0.42, *p* < 0.001), and gratitude was positively related to employees’ unethical pro-organizational behavior (model 5, β = 0.42, *p* < 0.001). To further examine the mediating effect of gratitude, we adopted the Bootstrap method as suggested by Hayes (2013). The results show that the indirect effect = 0.22, 95% CI [0.14, 0.32]. Thus, Hypothesis 2 was supported.

**FIGURE 1:**
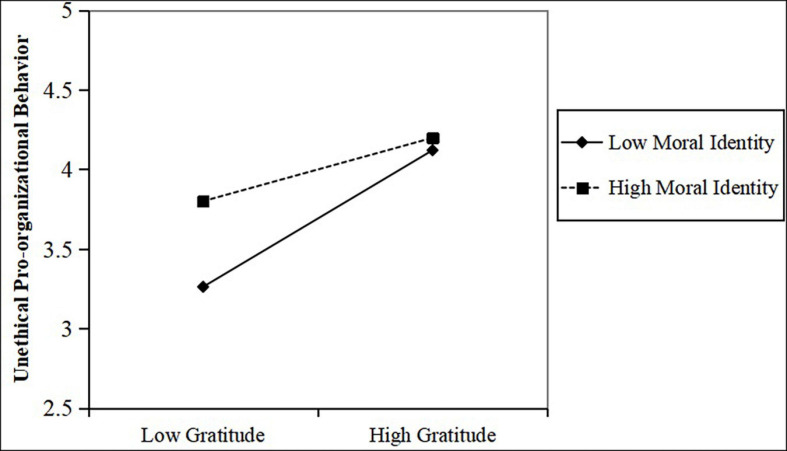
The moderating effect of moral identity.

Moreover, the interaction term had a significant effect on unethical pro-organizational behavior (model 7, β = −0.16, *p* < 0.01). We performed simple slope analysis to further examine the moderating effect of moral identity. As shown in Figure 1, when the moral identity was low, gratitude had a stronger effect on unethical pro-organizational behavior (*b* = 0.43, *p* < 0.001), and when the moral identity was high, the influence of gratitude on unethical pro-organizational behavior was weaker (*b* = 0.20, *p* < 0.05). Thus, Hypothesis 3 was supported.

In order to further test the moderated mediation effect, we adopted the Bootstrap method. Results showed that when moral identity was low (−1 SD), the indirect effect of leader forgiveness on unethical pro-organizational behavior *via* gratitude was 0.29, 95% CI [0.16, 0.44], when moral identity was high (+1 SD), the indirect effect of leader forgiveness on unethical pro-organizational behavior *via* gratitude was 0.13, 95% CI [0.05, 0.24]. In addition, the index of moderated mediation effect was −0.07, 95% CI [−0.14, −0.01], indicating that the moderated mediation effect was significant. Thus, Hypothesis 4 was supported.

## Discussion

Based on social exchange theory and affective event theory, the current research explores the mechanism and boundary condition of the influence of leader forgiveness on unethical pro-organizational behavior. Results show that gratitude mediates the relationship between leader forgiveness and unethical pro-organizational behavior, and moral identity moderates the relationship between gratitude and unethical pro-organizational behavior as well as the indirect influence of leader forgiveness on unethical pro-organizational behavior through gratitude.

### Theoretical Implications

First, this research contributes to leader forgiveness literature by revealing its possible negative consequences in organizations. Although previous research proposes leader forgiveness as a positive leader behavior, and finds it helps to improve employees’ physical and mental health, job satisfaction, and other pro-organizational behaviors. However, the previous research ignores the possible “dark side” of leader forgiveness. As researchers suggested that leader forgiveness helps to cultivate a culture that encourages being creative and taking risks, our study followed this line and established the link between leader forgiveness and unethical pro-organizational behavior, and found that leader forgiveness may lead employees to conduct pro-organizational behavior while violate ethical norms, thus providing new understanding about the consequences of leader forgiveness.

Second, drawing from the affective event theory, this research revealed the underlying mechanism process of the influence of leader forgiveness on unethical pro-organizational behavior by examining the mediating role of gratitude. In the past decades, management scholars have been working on opening the “black box” between variables, since this will help to understand how do these effects occur. By incorporating the mediating role of gratitude, our research showed that the influence of leader forgiveness on unethical pro-organizational behavior was realized through emotional change. In addition, previous research regarding employee’s unethical pro-organizational behavior mainly draws from the social exchange theory perspective, while our research, based on the affective event theory, further revealed that emotional change could also result in employees’ participating in unethical pro-organizational behavior. Thus, this study revealed the underlying mechanism process of the influence of leader forgiveness on unethical pro-organizational behavior from a new perspective.

Third, our research revealed the boundary conditions of the influence of gratitude on unethical pro-organizational behavior, and the indirect influence of leader forgiveness on unethical pro-organizational behavior *via* gratitude, by introducing moral identity as a moderating variable. Results showed that when moral identity is high, employees have more ability to identify ethical information, and to better regulate their behaviors to comply with ethical norms, thus will consider the long-term benefits of the organization and reduce their tendency to conduct unethical pro-organizational behavior. Our findings are consistent with previous research in the way that moral identity can effectively regulate and restrain individual’s unethical behaviors, and it can mitigate the “dark side” of leader forgiveness. Thus, our study helps to understand that when the influences of leader forgiveness and gratitude will be strengthened or weakened.

### Practical Implications

Our research also has some important practical implications regarding leader forgiveness and unethical pro-organizational behavior. First, our findings revealed that leader forgiveness could result in employees conducting unethical pro-organizational behavior, suggesting that organizational managers and leaders should be aware of the possible negative outcomes of conveying forgiveness in the workplace. Thus, organizations can design training programs to help leaders learn the potentially negative outcomes of forgiveness and educate them how to use forgiveness properly in the workplace.

Second, our research found that employees with high moral identity are less likely to conduct unethical pro-organizational behavior, suggesting that moral identity can effectively decrease the potentially detrimental impacts of leader forgiveness. Thus, organizations should consider to select and recruit individuals with high moral identity during the hiring process.

Third, given that unethical pro-organizational behavior is usually concealed and will harm organizations in the long term, organizations should keep an eye on this type of unethical behavior conducted by employees. And we encourage organizations to cultivate organizational cultures that condemn unethical behaviors, ethical leadership are also encouraged to act as role models to improve employees’ ethical standards to reduce unethical pro-organizational behavior.

### Limitations and Future Directions

This research also has several limitations. First, all data were collected from employees may cause common method variance. Future research could collect data from both employees and leaders, and conduct longitudinal research design to strengthen the causality. Second, our research was conducted in Chinese background, while employees from different cultural backgrounds may have different understandings about forgiveness. Thus, future research could cover employees from Western countries to increase the generality of our conclusions. Last, as we examined the moderating effect of moral identity on the relationship between gratitude and unethical pro-organizational behavior, results suggested that when gratitude was low, high moral identity employees conduct more unethical pro-organizational behavior, as compared with low moral identity employees. This counterintuitive result suggests that there may be other factors impact the linkages among gratitude, moral identity, and unethical pro-organizational behavior. Thus, a further in-depth research is encouraged to discuss under what circumstances high moral identity employees with low gratitude are more willing to engage in unethical pro-organizational behaviors.

## Conclusion

In this study, we developed our research model based on social exchange theory and affective event theory, and established linkages among leader forgiveness, gratitude, unethical pro-organizational behavior, and moral identity. Results show that leader forgiveness positively influences unethical pro-organizational behavior, and gratitude mediates the relationship between leader forgiveness and unethical pro-organizational behavior. The results also indicate that moral identity moderates the relationship between gratitude and unethical pro-organizational behavior, as well as the indirect influence of leader forgiveness on unethical pro-organizational behavior through gratitude.

The conclusions of this research are as follows: first, leader forgiveness will have a detrimental impact on organizations by encouraging employees’ unethical pro-organizational behavior. Secondly, leader forgiveness encourages employees’ unethical pro-organizational behavior through triggering their feelings of gratitude toward leaders, which suggests that employees may take advantages of leaders’ kindness and tolerance and participate in more risky behaviors. Finally, moral identity can effectively mitigate the detrimental impacts of leader forgiveness, as gratitude employees are less likely to conduct unethical pro-organizational behavior when they have high moral identity.

## Data Availability Statement

The raw data supporting the conclusions of this article will be made available by the authors, without undue reservation.

## Ethics Statement

Ethical review and approval was not required for the study on human participants in accordance with the local legislation and institutional requirements.

## Author Contributions

LL and YH designed and adopted the study and wrote the manuscript. LL and JL wrote the manuscript. All authors approved the submitted version.

## Conflict of Interest

The authors declare that the research was conducted in the absence of any commercial or financial relationships that could be construed as a potential conflict of interest.

## Publisher’s Note

All claims expressed in this article are solely those of the authors and do not necessarily represent those of their affiliated organizations, or those of the publisher, the editors and the reviewers. Any product that may be evaluated in this article, or claim that may be made by its manufacturer, is not guaranteed or endorsed by the publisher.
